# Elimination of mother-to-child transmission of HIV and syphilis in Cuba and Thailand

**DOI:** 10.2471/BLT.16.185033

**Published:** 2016-11-01

**Authors:** Naoko Ishikawa, Lori Newman, Melanie Taylor, Shaffiq Essajee, Razia Pendse, Massimo Ghidinelli

**Affiliations:** a Division of Communicable Diseases, World Health Organization Regional Office for the Western Pacific, United Nations Avenue, Manila, Philippines.; b United States Centers for Disease Control and Prevention, Phnom Penh, Cambodia.; c Department of Reproductive Health and Research, World Health Organization, Geneva, Switzerland.; d Department of HIV, World Health Organization, Geneva, Switzerland.; e Department of Communicable Diseases, World Health Organization Regional Office for South-East Asia, New Delhi, India.; f Department of Communicable Diseases and Health Analysis, Pan American Health Organization, Washington, United States of America.

Vertical transmission of human immunodeficiency virus (HIV) and syphilis can be effectively controlled through antenatal screening and treatment. However, each year there are still an estimated 150 000 cases of new paediatric HIV infections and 350 000 cases of congenital syphilis globally.^1^^,^^2^ The World Health Organization (WHO) has developed global health sector strategies for 2016–2021 on HIV and sexually transmitted infections that set the targets of achieving zero new HIV infections among infants by 2020 and less than or equal to 50 cases of congenital syphilis per 100 000 live births by 2030.^2^^–^^4^

WHO has issued a *Global guidance on criteria and processes for validation: elimination of mother-to-child transmission of HIV and syphilis*.^4^ To be validated by WHO as achieving elimination of the vertical transmission of HIV and syphilis, countries must meet three impact and five process targets, have a high-quality monitoring and surveillance system and respect basic human rights considerations, such as voluntary testing and treatment, equality and non-discrimination.^4^ Validation indicators specify that new paediatric HIV infections and congenital syphilis cases have to be less than or equal to 50 per 100 000 live births, and mother-to-child transmission rates of HIV have to be less than 5% in breastfeeding populations or less than 2% in non-breastfeeding populations for at least one year. When a country successfully meets the targets, it can submit a validation request and a national validation report to WHO. The report is reviewed by independent experts and an in-country assessment is conducted. This assessment involves a programmatic and health system review, in which high-level political commitment to elimination targets, strong maternal and child health and disease control programmes, reliable laboratory services, a robust health information system, compliance with human rights principles, gender equality and civil society engagement must all be demonstrated.

In 2015, Cuba became the first country in the world to receive validation from WHO for eliminating mother-to-child transmission of HIV and syphilis.^5^ Belarus and Thailand were validated for this achievement in 2016.^6^^,^^7^ Our in-country assessment of Cuba and Thailand showed that the two countries shared five common features that may have contributed to their success in achieving elimination.

First, both governments have maintained a strong commitment to prevent mother-to-child transmission of HIV and syphilis over a long period of time. Efforts to prevent congenital syphilis started in Cuba in the 1970s, and routine antenatal HIV testing was introduced in 1987.^8^ Similarly, congenital syphilis prevention programmes have been implemented in Thailand for over 30 years. Infant formula for HIV-exposed infants was introduced in Thailand in 1988 and antenatal HIV testing in 1993.^9^

Second, both governments successfully integrated prevention of mother-to-child transmission interventions into maternal and child health services free-of-charge. Routine HIV and syphilis testing for pregnant women and their partners is an essential element of both countries’ antenatal care packages. Mothers diagnosed with HIV or syphilis and their exposed infants are followed-up through health facilities linked to and supported by their communities.

Third, in both countries, infants exposed to HIV are monitored until the age of 18 months if breastfed. The health systems also track stillbirths, a common outcome of untreated maternal syphilis. Both countries have health information systems that provide timely and reliable data. These data are readily accessible to programme managers and health facilities for follow-up and programme monitoring.

Fourth, quality-assured diagnostic services for HIV and syphilis are accessible and provided free-of-charge. Laboratories use testing strategies and algorithms recommended by WHO.

Fifth, the assessment conducted with the representatives of regional civil society organizations confirmed that both countries comply with human rights and gender equality principles in their service provision. It was also noted that efforts to promote equity and inclusiveness are evident through provision of health care irrespective of citizenship or legal residence status.

These common features are indicative of the strong health systems that exist in both countries and reflect the steady progress they have made towards achieving universal health coverage. Total government health expenditure as a proportion of general government expenditure is high in both: 18% for Cuba and 23% for Thailand, above the 13% average of high-income countries.^10^ Government expenditure accounts for 95% of total health expenditure in Cuba and 85% in Thailand.^10^ Out-of-pocket contributions by patients in both countries are below 10%.^10^

The observation we have presented indicates that elimination of mother-to-child transmission of HIV and syphilis is possible when governments are committed to the health of their populations and to achieving universal health coverage.
